# Tracing Veterinary Pharmaceuticals through Regurgitated Pellets: A Noninvasive Monitoring Approach to Assess Exposure in Avian Scavengers

**DOI:** 10.1021/acs.est.6c01303

**Published:** 2026-06-18

**Authors:** Nelly Arlot, Marta Herrero-Villar, Rafael Mateo

**Affiliations:** † 69555 Institute for Game and Wildlife Research-IREC, CSIC-UCLM-JCCM, Ronda de Toledo s/n, Ciudad Real 13071, Spain; ‡ Institute for Environmental Assessment and Water Research (IDAEA-CSIC), Jordi Girona 18, Barcelona 08034, Spain

**Keywords:** veterinary pharmaceuticals, regurgitated pellets, avian scavengers, monitoring

## Abstract

Threatened avian scavengers can become highly exposed to pharmaceuticals when they feed on carcasses of medicated livestock. In this study, we assess the use of regurgitated pellets as a potential noninvasive method to monitor vulture exposure to pharmaceuticals. We analyzed pellets (*n* = 292) from Eurasian griffon vultures (*Gyps fulvus*) for 48 compounds commonly used in veterinary medicine in Spain as well as their persistence in such a matrix for up to one month in quasi-natural conditions. Samples were analyzed using liquid chromatography with electrospray ionization mass spectrometry (LC-ESI-MS/MS). We observed a high stability of most of the pharmaceuticals in the pellets. We detected veterinary pharmaceuticals in 44.2% of pellets, specifically 43.5% with antibiotics and 2% with NSAIDs (1.4% with both). Pellets containing only domestic livestock remains showed pharmaceuticals more frequently (48.2%, 91/189) than those containing wild ungulate remains (20.0%, 5/25). Pig was the main prey and remained associated with the presence of pharmaceuticals in pellets. The pellet monitoring of a supplementary feeding station supplied by a single farm revealed a temporal shift in the use of specific antibiotics. We highlight that the disposal of livestock carcasses to feed scavengers must be closely controlled because of potential risks posed by veterinary pharmaceutical residues, notably NSAIDs.

## Graphical abstract

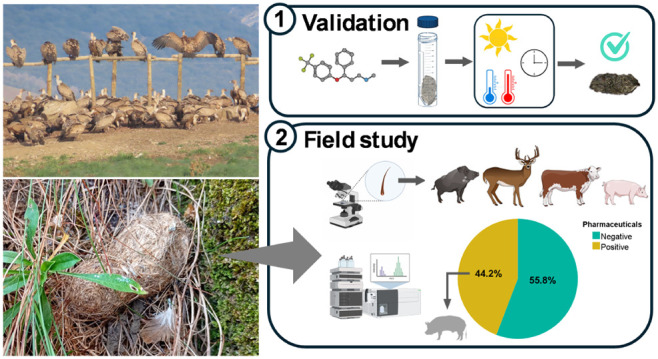

## Introduction

Wildlife exposure to veterinary pharmaceutical products can occur via ingestion of contaminated water and food, but the carcasses of medicated livestock are the most direct and important source for avian scavengers and can even pose significant risks for their conservation.^1^ One of the most telling examples occurred in southern Asia, where the nonsteroidal anti-inflammatory drug (NSAID) diclofenac used in cattle and other livestock drastically reduced the population of *Gyps* vultures (*G. bengalensis*, *G. indicus*, and *G. tenuirostris*) by 99%, leading them to near extinction.^2–5^

Despite studies showing the impact of diclofenac on *Gyps* vulture populations in Asia, this anti-inflammatory drug was authorized for veterinary use in Spain in 2013, and it caused in 2020 the first documented death of a cinereous vulture (*Aegypius monachus*).^6,7^ Although the impact of diclofenac use on vultures in Spain has not been as catastrophic as in South Asia until now, surveillance must be maintained because diclofenac use could become more frequent in the future.^6^ Moreover, other NSAIDs such as ketoprofen, carprofen, aceclofenac, flunixin, and nimesulide have been recognized as highly toxic to vultures too and, therefore, should also be monitored as they have the potential to seriously impact vulture populations.^8–13^ Flunixin poisoning has been reported in vultures in Spain.^12,14^ In addition to NSAIDs, veterinary drugs used to euthanize animals such as barbiturates cause lethal poisonings in avian scavengers when carcass disposal has not been properly performed.^15,16^ Moreover, recent studies done in Spain have revealed the presence of veterinary pharmaceuticals, mostly antibiotics, in 54.1% of livestock carcasses supplied at supplementary feeding sites (SFSs) for vultures and in the tissues of 51.7% of avian scavengers found dead, and in the plasma of 28.5% of avian scavengers trapped alive.^17^

Modeling approaches have assessed that contamination with lethal levels of diclofenac of a very small proportion (0.13% to 0.75%) of ungulate carcasses can lead to a decline of ∼30% per year of scavenger populations.^18^ As dramatic consequences can unfold rapidly, consistent and thorough monitoring is imperative to evaluate and manage the toxicological risks experienced by vulture populations. Given the difficulty of sampling scavenged carcasses or live vultures, an easier alternative could be the collection of regurgitated pellets for noninvasive analysis. These have been used for assessing the ingestion of plastics or lead ammunition.^19–24^ Chemical analyses of regurgitated pellets have also been conducted to assess the risk posed by rodenticide anticoagulants to barn owls (*Tyto alba*).^25–27^ To date, no study on veterinary pharmaceuticals in scavenger pellets has been performed, but this technique could facilitate extensive studies without the use of more invasive and time-consuming practices. The aim of this study was (1) to study the stability of 44 pharmaceuticals of interest in regurgitated pellets of vultures exposed to natural UV light and temperature variations; (2) to apply an analytical method to detect pharmaceuticals in pellets from wild vultures collected near SFSs in different regions of Spain to determine the prevalence of exposure; and (3) to establish associations between the presence of pharmaceuticals and the vulture’s diet by the identification of the type of prey (domesticated, wild, or mixed) present in the pellets.

## Materials and Methods

### Study Area

The study was conducted in various provinces of Central and Northeastern Spain, specifically within the autonomous communities (regions) of Aragón, Castilla-La Mancha, Castilla y León, La Rioja, and Navarra. These regions were chosen due to the known locations of numerous vulture roosting sites, which facilitated field efforts through their GPS coordinates. Selection criteria included proximity to intensive livestock farms, particularly those focusing on pig farming (e.g., Aragón, Soria province in Castilla y León) or poultry farming (Navarra), as well as the presence of numerous SFSs. This environment led to a significant influx of vultures and enabled the collection of pellets at roosting sites, increasing the likelihood of containing pharmaceutical residues. Sampling of less intensively managed regions such as Guadalajara (Castilla-La Mancha) is also carried out to compare toxicological loads between sites.

### Sample Collection

Pellet sampling (*n* = 292) was carried out between 2021 and 2024 at several roosting sites of Eurasian griffon vultures (*Gyps fulvus*) in Castilla y León (5 sites), Aragón (8 sites), Castilla-La Mancha (3 sites), Navarra (2 sites), and La Rioja (1 site), located near SFSs (Figure 1; Table S1). The species using these SFSs were mainly griffon vultures, but cinereous vultures, Egyptian vultures (*Neoprhon percnopteurs*), and bearded vultures (*Gypaetus barbatus*) also visited these feeding sites in lower numbers. All samples were deposited into Ziploc bags or polypropylene tubes and frozen at −20 °C until analysis. The pellets collected for pharmaceutical analysis were those with a high moisture content and compact shape, as they were associated with greater freshness and minimal exposure time to ambient air and weather conditions. In contrast, very dry pellets were excluded because they were associated with prolonged exposure to outdoor conditions and a likely loss of pharmaceutical load due to degradation.

**Figure 1:**
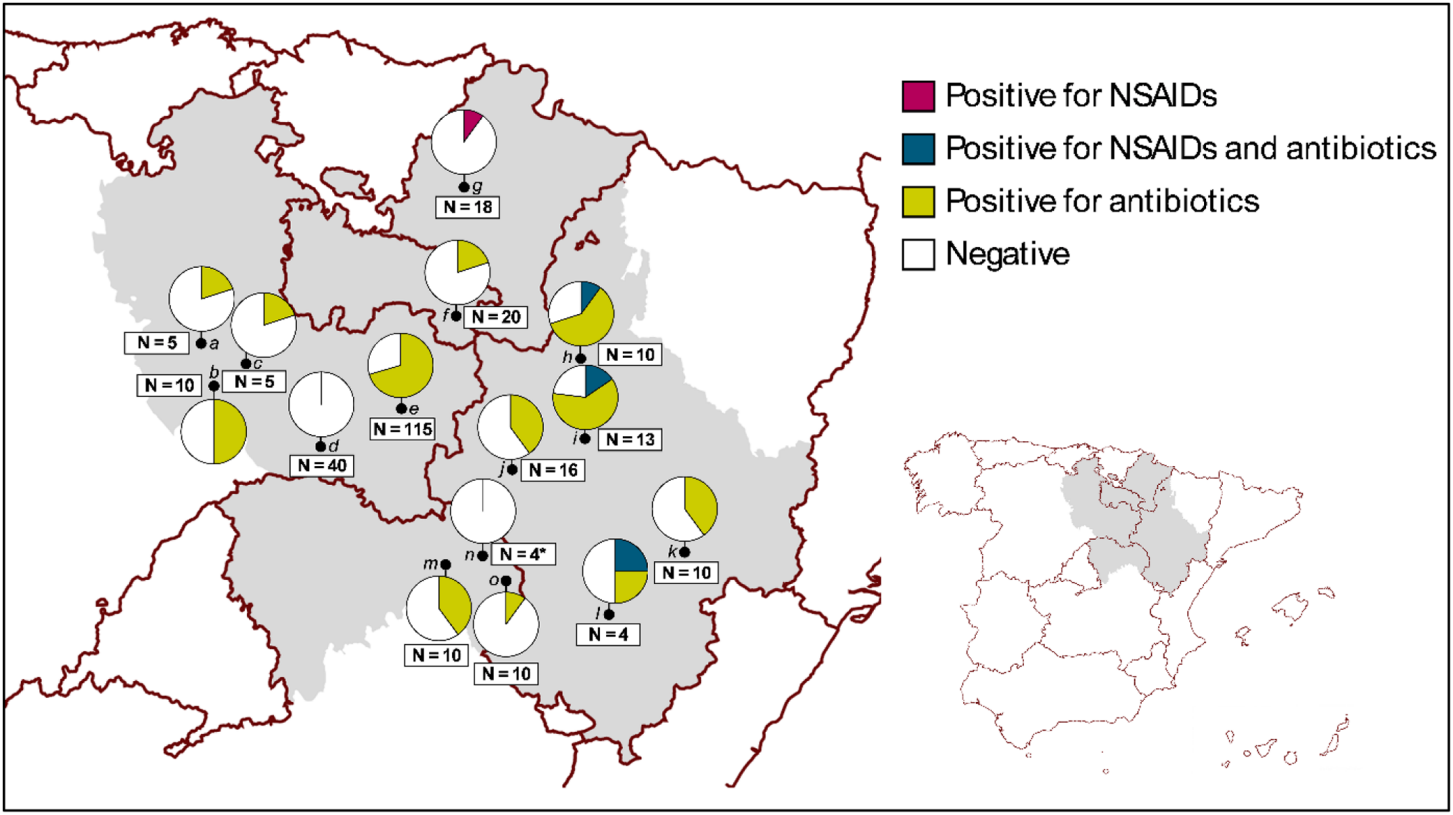
Map of the study area with the sites of interest and their respective prevalence of pellets positive to pharmaceuticals. The sites are as follows: a: Nebreda, b: Huerta del Rey, c: Caleruega, d: Berlanga de Duero, e: Los Rábanos, f: Igea, g: Murillo, h: Illueca, i: Morata de Jalón, j: Calatayud, k: La Mata de los Olmos, l: Perales de Alfambra, m: Ventosa, n: Peralejos de las Truchas, and o: Pobo de Dueñas.

A specific sampling was conducted at the Los Rábanos site (Castilla y León) in 2024, where we collected pellet samples during two visits within 1 week in winter. This roosting site of interest is located near a SFS in an area with a high density of intensive pig farms. During the first visit, the freshest pellets were collected as done at other sites, and the area was subsequently cleaned to remove debris and older pellets. This allowed the identification and collection of new pellets aged only a few days during the second visit. This targeted sampling enabled the assessment of short-term variability in the presence of pharmaceuticals in the same SFS and whether pellet freshness is a relevant factor influencing pharmaceutical quantification. This adds to regular sampling conducted during previous years in order to study seasonality and long-term variability between samplings.

### Assessment of the Stability of Pharmaceuticals in Pellets

The 48 compounds selected for testing (Table S2) represent the main nonpolar pharmaceutical families approved for veterinary use in Spain, with the exception of 2 antibiotics (nalidixic acid and sulfamethoxypyridazine) and 4 NSAIDs (hydroxyethyl salicylate, indomethacin, naproxen, and nimesulide), which are still included in the method either because they have only recently been revoked or because they are still used as veterinary treatments in other regions of the world.^28^ The final list included 12 NSAIDs, 32 antibiotics, 3 antiparasitic drugs, 1 anticholinergic drug, and caffeine (used as a chemical marker of urban waste consumption).^17^ The stability test was performed with pellet material spiked with 44 of these compounds and exposed to outdoor conditions. To achieve this, 1 g of a pool of pellets free of any pharmaceuticals was used, spiked with 200 ng of the pharmaceutical mix detailed previously, and placed in transparent polypropylene tubes. These tubes were then left open outside for 24 h (*n* = 5), 48 h (*n* = 5), 96 h (*n* = 5), 1 week (*n* = 5), 2 weeks (*n* = 5), and 1 month (*n* = 5) before being sealed and stored in a freezer at −20 °C until analysis. They were compared to blank samples (nonspiked pellet pool, *n* = 5) and samples spiked with the same pharmaceutical load (*n* = 5) and stored in the freezer throughout the experiment, serving as background and spiked reference samples, respectively, for the calculation of stability. This type of exposure method enables us to assess the effect of time as well as UV light and temperature fluctuations between day and night. No rain was reported during the study performed in July 2023, but high temperatures were commonly recorded during the day (>35 °C). An extraction and quantification of the pharmaceuticals were then carried out according to the subsequent protocol.

### Pharmaceutical Analysis

The pharmaceutical extraction technique was adapted from Herrero-Villar et al. (2023).^17^ We first took measurements of the entire pellets (length, width, freshness, mass) collected in the field and selected 0.5 g from the inner part of each one (on average, representativeness accounts for 5% of the total weight of each pellet), as we considered this to be the best-preserved part, less likely to have had its components affected by external elements. In the case of the samples used for the stability assay, the whole sample (1 g) used in the tests was analyzed. The pellet sample was homogenized in a 50 mL Falcon tube to which 50 μL of internal standard (IS) (enrofloxacin-*d5* and flunixin-*d3* at 10 ng/μL) was added. Then, 20 mL of a methanol and Milli-Q water solution (75:25) acidified with 0.1% formic acid (solvent) was incorporated. This mixture was vortexed using a Multi-TX5 vortex mixer for 5 min, then sonicated in an ultrasonic bath for 5 min, and finally centrifuged at 4700 rcf for 5 min. The entire supernatant was then transferred to a 50 mL Falcon tube (F1). The remaining pellet sample was subjected to these steps a second time for a double pharmaceutical extraction, which means that 20 mL of solvent were added again, vortexed, sonicated, and centrifuged for 5 min each time. The new supernatant (F2) was collected and mixed up with the F1 supernatant, and 7 mL of this mixture were then transferred to a 15 mL Falcon tube containing 150 mg of C18 and 900 mg of MgSO_4_ (QuEChERS dispersive kit: 5982–4956, Agilent) to remove biological matrix interferences, including hydrophobic substances and proteins. This new mixture was again subjected to 5 min of vortexing, sonication, and centrifugation (at 2500 rcf). We obtained 1 mL of the QuEChERS cleanup supernatant and filtered this extract into a 2 mL HPLC vial. The extracts obtained were immediately analyzed or were stored at −20 °C until analysis. Following the same protocol, a blank sample as well as three spiked samples were analyzed alongside each sample batch (∼50 pellets), using pellets without pharmaceutical products. The spiked loads consisted of 25, 50, and 100 μL of a pharmaceutical mix solution containing all 48 previously listed compounds (10 ng/μL) and 50 μL of the IS solution.

The extracts were then analyzed using liquid chromatography coupled with electrospray ionization mass spectrometry (LC-ESI-MS/MS) utilizing an Agilent 6470 LC connected to an Agilent G7104A triple quadrupole MS. Chromatographic separation was conducted using an Agilent Poroshell-120EC-C18 column (2.1 × 150 mm, 2.7 μm). The chromatography conditions were as follows: a flow rate of 0.3 mL/min, column temperature set at 40 °C, and gradient elution with solvent A (0.1% formic acid in Milli-Q water) and solvent B (methanol). Initially, the conditions were set to 98% solvent A and 2% solvent B for 1 min, followed by a linear gradient over 9 min to 75% A and 25% B. This was followed by a 5 min linear gradient to 100% B, which was maintained for 2 min before returning over 7 min to the initial conditions, which were then maintained for 4 min before the next sample injection. The injection volume was 10 μL, and vials were kept at 4 °C in the autosampler. For the triple quadrupole MS, parameters included a gas temperature of 300 °C, a gas flow rate of 7 L/min, a sheath gas temperature of 350 °C, a sheath gas flow rate of 11 L/min, and a nebulizer pressure of 45 psi. Molecular weights for precursor ions and the two main fragmentation ions for compounds were analyzed in multiple reaction monitoring mode with positive and negative ionization. Quantification was conducted using the most abundant fragment ion, employing a fragmentation voltage of 50–500 V and a capillary voltage of 3500 V.

Calibrations were carried out using diluted working solutions prepared from standard solutions at 1 mg/mL for each compound. The mixed working solutions were then stored at 4 °C until use. To take into account the expected effects of the matrix, a calibration curve was prepared on a matrix extract obtained from blank pellets (matrix-matched calibration). The calibration vials were prepared at concentrations of 6.25, 12.5, 25, 50, 100, and 200 ng/mL from the pharmaceuticals mix, with matrix-matched extract and 50 μL of IS (10 ng/mL) to end up with a final volume of 1 mL. The validation of the analytical method included the calculation of the accuracy and precision obtained with the spiked samples (% recovery ± RSD). An acceptable recovery range was considered to be between 70% and 120%, with the RSD below 20%.^29^ We obtained recovery values between 77% for marbofloxacin and 112% for doramectin (Table S2). Only amoxicillin and oxytetracycline showed recovery values out of the specified range, and, if present in samples, these results should be considered with caution. Regression coefficients (R^2^) in matrix-matched calibration curves were between 0.9765 for ivermectin and 0.9973 for hydroxyethyl salicylate. Limits of quantification (LOQs) were between 0.001 and 0.002 ng/g (for sulfamethoxypyridazine) and 5.666–18.888 ng/g (for indomethacin) in pellets (Table S2). These values were established as 10*SD/S, where SD is the standard deviation of the response of a standard at the lowest identifiable concentration and S is the slope of the calibration curve of the analyte.^30^

### Prey Identification in Pellets

A subsample was taken from each pellet for optical microscopic identification of the main prey present in each regurgitation to study their association with the presence of pharmaceuticals. Hair identification was based on the key and method developed by De Marinis and Asprea (2006) based on hair medulla and cuticle features of wild and domestic ungulate mammals.^31^

### Data Analysis

We generated a time-series plot for each pharmaceutical compound included in the stability test to evaluate its persistence in vulture pellets. These plots depict the mean concentration at each time point, allowing for visual inspection of trends and stability throughout the study period.

In the case of the pellets collected in the field, we used Generalized Linear Models (GLMs) with a binomial distribution (presence/absence of pharmaceutical) to ensure the robustness of the analysis as antibiotics and NSAIDs are commonly administered together in veterinary practice. We also included a logit link function to determine the specific type of prey (pig, poultry, sheep, goat) or prey categories (domestic livestock, wild ungulates, or mixed) associated with pharmaceutical occurrence. Year (2021, 2022, 2024), season (winter, spring, and summer), and regions (Aragón, Castilla-La Mancha, Castilla y León, La Rioja, and Navarra) were also included in the models (Table S4). The site was not included as a random or nested effect because it was closely associated with the type of species found in pellets (χ^2^ = 65.8, d.f. = 28, *p* < 0.001), and our hypothesis focused on diet as the primary driver of pharmaceutical exposure. Additional GLMs were performed using the log-transformed concentration of the sum of pharmaceuticals detected in the pellet with a normal distribution and an identity link function, incorporating the same descriptors used in the prevalence binomial models. Model stepwise selection was based on the significance of the effect of each factor and Akaike’s Information Criterion corrected for small sample size (AICc), including all the cited descriptors (Table S5).^32^ Significance was established at *p* ≤ 0.05. The statistical analysis was performed using IBM SPSS Statistics 28.0.

## Results and Discussion

This is the first study using regurgitated pellets to assess exposure to veterinary pharmaceuticals in avian scavengers. Residues of antibiotics and two NSAIDs (flunixin and meloxicam) were detected and quantified in vulture pellets collected in Spain between 2021 and 2024. The relative stability of most of the studied pharmaceuticals in the regurgitated pellets (more than 50% of the spiked amount being detected up to 14 days under outdoor environmental conditions) supports the suitability of this noninvasive method for large-scale monitoring programs with a lower cost or effort than livestock carcass or live vulture sampling.

### Assessment of the Stability of Pharmaceuticals in Pellets

The tested pharmaceuticals were detectable for up to 28 days in spiked pellets maintained under outdoor environmental conditions. Of the 44 pharmaceuticals tested here, 4 (1 antibiotic: chloramphenicol and 3 NSAIDs: carprofen, indomethacin, and phenylbutazone) showed a low signal at time 0 and were excluded from the stability test. Particularly, antimicrobials were more stable than NSAIDs and scopolamine. The most relevant pharmaceuticals to be monitored in field studies (i.e., antimicrobials and NSAIDs) were detectable until 2 weeks with concentrations above 50% of the initially spiked amount (Figure 2; Table S3).

**Figure 2:**
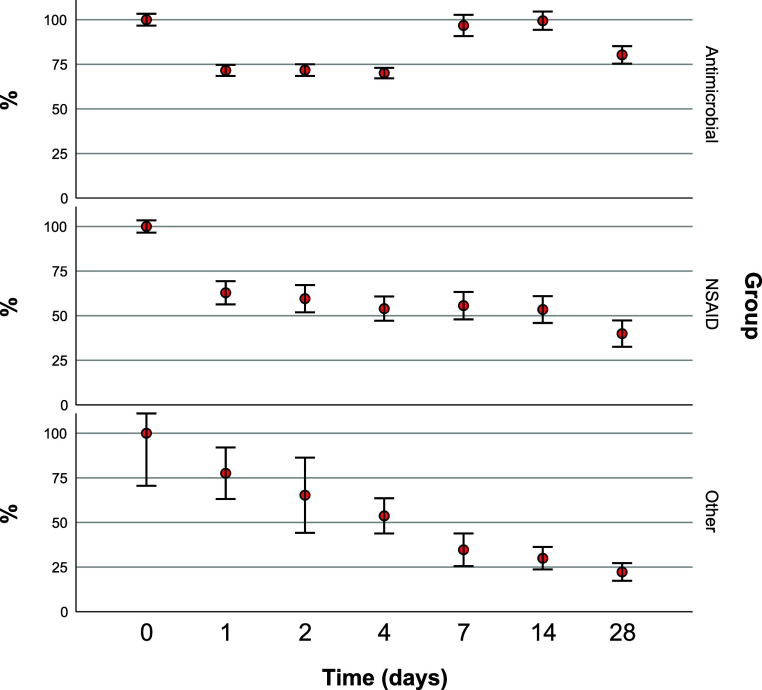
Percentage (mean with 95% CI) of pharmaceuticals (by functional groups) detected in spiked pellets up to 28 days of storage at outdoor environmental conditions in comparison to the amount detected at time 0. See Table S3 for the values of specific pharmaceuticals. Other refers to scopolamine.

The persistence of pharmaceuticals in pellets could be influenced by several factors like the time since regurgitation or the environmental conditions (i.e., sunlight, temperature, rainfall), as observed with other types of chemicals in other animal excreta (i.e., anticoagulant rodenticides in the feces of red foxes (*Vulpes vulpes*)).^33^ In the present study, the pellets spiked with pharmaceuticals were subjected to significant UV exposure and consistently high temperatures throughout the month. These conditions likely accelerated the degradation of some compounds (i.e., NSAIDs and scopolamine) while not affecting others (i.e., most of the antimicrobials). The fluctuations in antimicrobial levels, including occasional increases, may be explained by slight changes in the matrix composition during maintenance under outdoor natural conditions that can affect mass spectrometry even with a matrix-matched calibration prepared with fresh pellet extract. The decrease in certain components, such as organic matter, may have influenced the matrix effect on compound ionization efficiency. Other studies have shown that changes in matrix composition can significantly affect analytical results.^34^ Several methods can be employed to mitigate matrix effects, such as selective extraction, through sample cleanup postextraction or enhancing chromatographic separation. However, these methods are not always suitable as they might cause analyte loss and extend analysis times.^35^ Effective strategies mentioned in the literature include using appropriate calibration techniques like external calibration with matrix-matched samples and internal standards, as we used here, but also calibrations by standard addition or extract dilution to reduce matrix effects on analyte detection.^36–38^ Nevertheless, this method remains feasible in the field, as most compounds are still detectable after a month in pellets not exposed to rain. This finding is crucial for long-term field studies where immediate sample collection is not always possible. However, some compounds seem to be rapidly degraded in pellets exposed to environmental conditions (i.e., UV light, temperature, humidity), like carprofen, chloramphenicol, indomethacin, and phenylbutazone. If these compounds were of special interest, it would be preferable to remove old pellets under the roosts 2 days before sampling to collect only fresh pellets for pharmaceutical analysis. In the case of amoxicillin and oxytetracycline, these antibiotics should be tested using higher levels (as seen in the quantification of the samples) to perform this same experiment.

### Presence of Pharmaceuticals in Pellets

Veterinary pharmaceuticals were detected in 44.2% of pellets (*n* = 129), with 43.5% containing antibiotics (*n* = 127) and 2.1% containing NSAIDs (*n* = 6) (1.4% showed both antibiotics and NSAIDs, *n* = 4) (Table 1). Herrero-Villar et al. (2023) detected pharmaceuticals in 54.1% of livestock carcasses and in 53.5% of griffon vulture livers, which is the dominant species in SFSs.^17^ Moreover, the prevalence of pharmaceuticals in pellets was higher than that found by Herrero-Villar et al. (2023) in plasma samples of griffon vultures, where only 28.9% tested positive for veterinary pharmaceutical residues.^17^ This difference is likely due to the close similarity of regurgitated pellets to the main prey in the griffon vulture diet (i.e., domestic livestock). On the contrary, pharmaceuticals in blood undergo rapid metabolization, which may reduce detectability.^39^ No unauthorized veterinary pharmaceuticals were detected in this study. Out of the 292 analyzed pellets, 162 contained no pharmaceutical products. Among the rest, 64 pellets contained 1 pharmaceutical, 32 contained 2, 20 contained 3, 6 contained 4, 2 contained 5, 5 contained 6, and one pellet contained residues from 8 different pharmaceuticals. The pellets with the greatest pharmaceutical cocktails were from Morata (Aragón) and Igea (La Rioja).

**Table 1:** Veterinary Pharmaceutical Positive Pellets (N+), Prevalence (%+), and Concentrations Detected in Pellets of Eurasian Griffon Vulture (*Gyps Fulvus*) (*n* = 292) Collected at Roosting Sites Near Supplementary Feeding Stations in Spain

				Concentration (ng/g)	
Chemical family	Compounds	N+	%+	Mean ± SD	Max
**NSAIDs**	Flunixin	6	2.1	14.8 ± 6.3	45.0
	Meloxicam	1	0.3	2.32	2.32
**Antibiotics**					
Lincosamides	Clindamycin	1	1.0	2.27	2.27
	Lincomycin	42	14.4	3.94 ± 1.1	34.8
Macrolides	Erythromycin	1	0.3	22.9 ± 0.8	23.0
	Tilmicosin	7	2.4	47.0 ± 29.6	222.4
	Tulathromycin	2	0.7	128.8 ± 74.9	203.7
Phenicols	Florfenicol	1	0.3	122.8	122.8
Pleuromutilin	Tiamulin	6	2.1	1.34 ± 0.3	2.71
Quinolones	Ciprofloxacin	35	12.0	3.93 ± 0.86	19.0
	Enrofloxacin	66	22.6	30.3 ± 9.3	548.0
	Flumequine	1	0.3	1.32	1.32
	Marbofloxacin	8	2.7	7.47 ± 2.0	16.0
Sulfonamides	Sulfadiazine	19	6.5	34.6 ± 20.9	392.3
	Sulfadoxine	5	1.7	8.12 ± 3.2	20.4
	Sulfamethoxazole	1	0.3	6.76	6.76
Tetracyclines	Chlortetracycline	7	2.4	13,615 ± 13,346.7	93,694.5
	Doxycycline	8	2.7	193.1 ± 129.6	1092.5
	Oxytetracycline	17	5.8	4,285 ± 15,206.3	63,179.7
	Tetracycline	1	0.3	977.2	977.2
Trimethoprim	Trimethoprim	22	7.5	24.4 ± 10.5	242.2
**Total**		129	44.2		

Regarding specific NSAIDs, flunixin (*n* = 6) and meloxicam (*n* = 1) were the only ones detected. Flunixin and meloxicam are the most frequently reported NSAIDs in the literature on Spanish scavengers; even so, the prevalence in our study is lower than in previous studies. Specifically, 2% of pellets tested positive compared to 10.8% in livestock carcasses, 3.6–6.0% in avian scavenger tissues, and 4.7% in vulture plasma samples.^14,17^ Meloxicam and tolfenamic acid are currently the only NSAIDs recognized as safe for vultures, so the presence of meloxicam in a pellet does not pose a significant toxicological risk. Flunixin was detected in six samples, reaching a concentration of 44.97 ng/g, specifically in Los Rábanos SFS, suggesting foraging on highly treated carcasses. Flunixin concentration in livestock tissues supplied at SFSs ranges between 1.7 and 24,500 ng/g, although the relationship between concentrations in ingested tissues and those subsequently detected in pellets remains unknown.^14,17^ The potentially toxic dose for *Gyps* vultures of flunixin is 1–5 mg/kg body weight, which in a griffon vulture of 7 kg of body weight and a daily intake of 0.5 kg may correspond to a prey item with 14 mg/kg.^11^ In any case, its presence at three sites highlights that this pharmaceutical, which is among the most toxic, is being supplied at feeding stations and thus enters the avian scavenger food chain in Spain.

For antibiotics, the most frequently detected were enrofloxacin (*n* = 66), lincomycin (*n* = 42), ciprofloxacin (*n* = 35), trimethoprim (*n* = 22), sulfadiazine (*n* = 19), and oxytetracycline (*n* = 17) (Table 1). The pharmaceuticals most commonly found together in one pellet were ciprofloxacin and enrofloxacin (*n* = 33, occurring frequently together because ciprofloxacin is a metabolite of enrofloxacin), lincomycin and enrofloxacin (*n* = 12), and sulfadiazine with trimethoprim (*n* = 12) (which are commonly formulated together in the pharmaceutical product). Here, we detected several quinolones (ciprofloxacin, enrofloxacin, flumequine, and marbofloxacin) that had been detected before in livestock and vultures. The prevalence of enrofloxacin (22.6%) is similar to the prevalence observed in livestock carcasses (21.1%).^30^ This prevalence was higher than the values reported in the plasma of wild adult griffon vultures (11.3–15.1%) but lower than the prevalence found in the plasma of vulture nestlings (31.3–100%).^17,30,40–44^ Enrofloxacin and ciprofloxacin concentrations in pellets (mean of 30.26 ng/g and 3.93 ng/g, respectively) were lower than the concentrations detected in livestock tissues supplied at SFSs (493–955 ng/g and 381–406 ng/g, respectively) and in the liver of griffon vultures (244 ng/g and 37.5 ng/g, respectively) in Spain.^17^ The other two quinolones, flumequine, had been detected before in the liver of Egyptian vultures (in 31.6%), and marbofloxacin had been detected in tissues of livestock carcasses (9.4%) supplied at SFSs.^17^ Although quinolone concentrations in livestock are below toxicity thresholds for birds, some studies have reported yeast infections in the oral cavity of avian scavengers linked to antibiotic exposure, potentially affecting the balance of their oral microbiota.^30,42,45,46^

Penicillins and tetracyclines are by far the most commercialized antibiotic classes in the EU for veterinary use in food-producing animals (EMA, 2022).^47^ In this study, four tetracyclines (chlortetracycline, doxycycline, oxytetracycline, and tetracycline) were detected. The prevalence, ranging from 0.3 to 5.8%, is lower than what has been reported in the literature, with detected prevalence in the plasma of Spanish griffon vultures reaching up to 11.4%.^17,44^ An unexpected finding was the particularly high concentrations of oxytetracycline, reaching 63,180 ng/g. High levels of oxytetracycline have previously been detected in sheep tissues in Portugal, reaching up to 1,452.7 ng/g, which is still 900 times lower than the maximum level we detected.^48^ However, as previously mentioned, when establishing our method, oxytetracycline showed recovery values outside the specified range, indicating that these levels may have been inaccurately quantified by our current method, even though precautions have been taken in view of this disclaimer to select only real positives. Nevertheless, these results should be interpreted with caution and confirmed through additional analysis. Chlortetracycline was also detected, with an average high concentration of 13,615 ng/g (up to 93,693 ng/g), without comparable data in the same context. The high persistence of these types of antibiotics in livestock tissues, coupled with the potential accumulation of these widely used compounds when scavenging multiple carcasses, may help explain the high detection rates in pellets in this study.^49^

Additionally, we detected three macrolides (erythromycin, tilmicosin, and tulathromycin), three sulfonamides (sulfadiazine, sulfadoxine, and sulfamethoxazole), one pleuromutilin (tiamulin), and trimethoprim. A previous study reported the presence of other antibiotic families, including macrolides, lincosamides, sulfonamides, and trimethoprim, in both the liver and plasma of avian scavengers in Spain.^17^ However, this is the first time that tiamulin, sulfamethoxazole, and chlortetracycline have been detected in such a context regarding the monitoring of avian scavenger exposure to pharmaceuticals. Exposure to antibiotics at the levels detected in carrion has not yet been linked to acute toxic effects in avian scavengers, and potential adverse effects due to chronic exposure to pharmaceutical cocktails in the long run are still to be elucidated.^30,42,45,46^

### Determinants of Pharmaceutical Presence in Pellets

Pigs were the most common dietary item found in vulture pellets, constituting 61.6% (*n* = 180) of occurrences. Goats were the second most common, with 25.7% (*n* = 75), followed by deer species (roe deer and red deer) with 33.6% (*n* = 98), poultry with 24.3% (*n* = 71), sheep with 20.5% (*n* = 60), and wild boar with 2.7% (*n* = 8). The pellets containing only domestic animals represent 64.7% of the samples, those with only wild animals accounted for 8.6%, and those containing both types make up 26.7% of the samples. The prevalence of pharmaceuticals in pellets containing only domestic livestock remains was 48.2% (91/189), and it was significantly higher than in the pellets containing only wild ungulate remains, with 20.0% (5/25) prevalence (Wald’s χ^2^ = 7.582, d.f. = 1, p = 0.014; model 1A, Table S4). Pellets containing a mixture of domestic livestock and wild ungulate remains showed a prevalence of 42.3%, similar to those containing only domestic livestock. NSAIDs were not detected in pellets containing only wild ungulate remains. At the prey species level, there was a significant positive association between the detection of pharmaceuticals and the presence of pig remains in the pellet (Wald’s χ^2^ = 15.244, d.f. = 1, *p* < 0.001; model 2A, Table S4).

In terms of pharmaceutical concentrations, the presence of pig remains was not a significant descriptor, but this concentration was negatively associated, close to the significance level, with the presence of sheep remains (Wald’s χ^2^ = 3.768, d.f. = 1, p = 0.052; model 3 (lower AICc than the model with region but without sheep presence, Table S4). The consumption of domestic livestock, and particularly pig, was associated with the presence of pharmaceuticals in the regurgitated pellets, as observed before with the analysis of plasma of griffon vultures captured at SFSs supplied with different types of animal carcasses.^17^ According to pharmacokinetic data, some frequently used veterinary drugs, such as quinolones, tend to accumulate in pig tissues for extended periods (up to 240 h in the liver and kidney) compared to other species like sheep, rabbits, or chickens.^49^ Regarding NSAIDs, flunixin was detected in pellets from the three sites where the presence of poultry feathers in pellets was among the highest (Murillo, which is associated with intensive poultry farms, Morata, and La Mata). This association can be supported by the detection of flunixin in poultry carcasses by Herrero-Villar et al. (2023).^17^

Regarding the sampling location, the highest proportion of pharmaceutical-positive pellets was found in Morata (*n* = 13) (Aragón) with 76.9%, while the sites with the lowest proportions were Berlanga (*n* = 40) (Castilla y León) and Guadalajara (*n* = 4) (Castilla-La Mancha), each with 0%. Across the 16 sampled sites, the average prevalence was 34.7% (Figure 1). Five sites out of 15 (Morata, Los Rábanos, Illueca, Caleruega, and Perales) exhibited a prevalence of 50% or higher, all of which were in Aragón or Castilla y León. Therefore, the prevalence of pharmaceutical exposure differed between regions (Wald’s χ^2^ = 8.097, d.f. = 1, p = 0.004; model 1A, Table S4) and was higher in Aragón and Castilla y León than in the other sites. This effect of the region was observed when the diet factor was included in the model, suggesting that variability between regions may respond to differences in carcass management at SFSs or in the use of pharmaceuticals on farms in these autonomous communities. In terms of pharmaceutical concentration, the effect of the region was also significant (Wald’s χ^2^ = 9.145, d.f. = 1, p = 0.002; model 3, Table S4), with the highest concentrations in Castilla y León and Aragón.

The proportion of positive pellets did not differ significantly among years or seasons in the models comparing types of prey. However, the repeated sampling at Los Rábanos (supplied by a pig farm) has shown within-site variability in the detected antibiotics, and the variable year was present in the best model according to AICc values and was significant in the specific model with pig presence (Table S4). Enrofloxacin was the most frequently detected in March 2021, February 2022, and July 2022, but lincomycin was afterward the most frequently detected in December 2022 and February 2024 (Figure 3). Interestingly, the results of the two consecutive samplings before and after cleaning the old pellets and remains under the roosting sites in February 2024 showed similar results in terms of antibiotic presence, which may reflect the correct selection of recent pellets in single visits based on their shape, consistency, and humidity.

**Figure 3:**
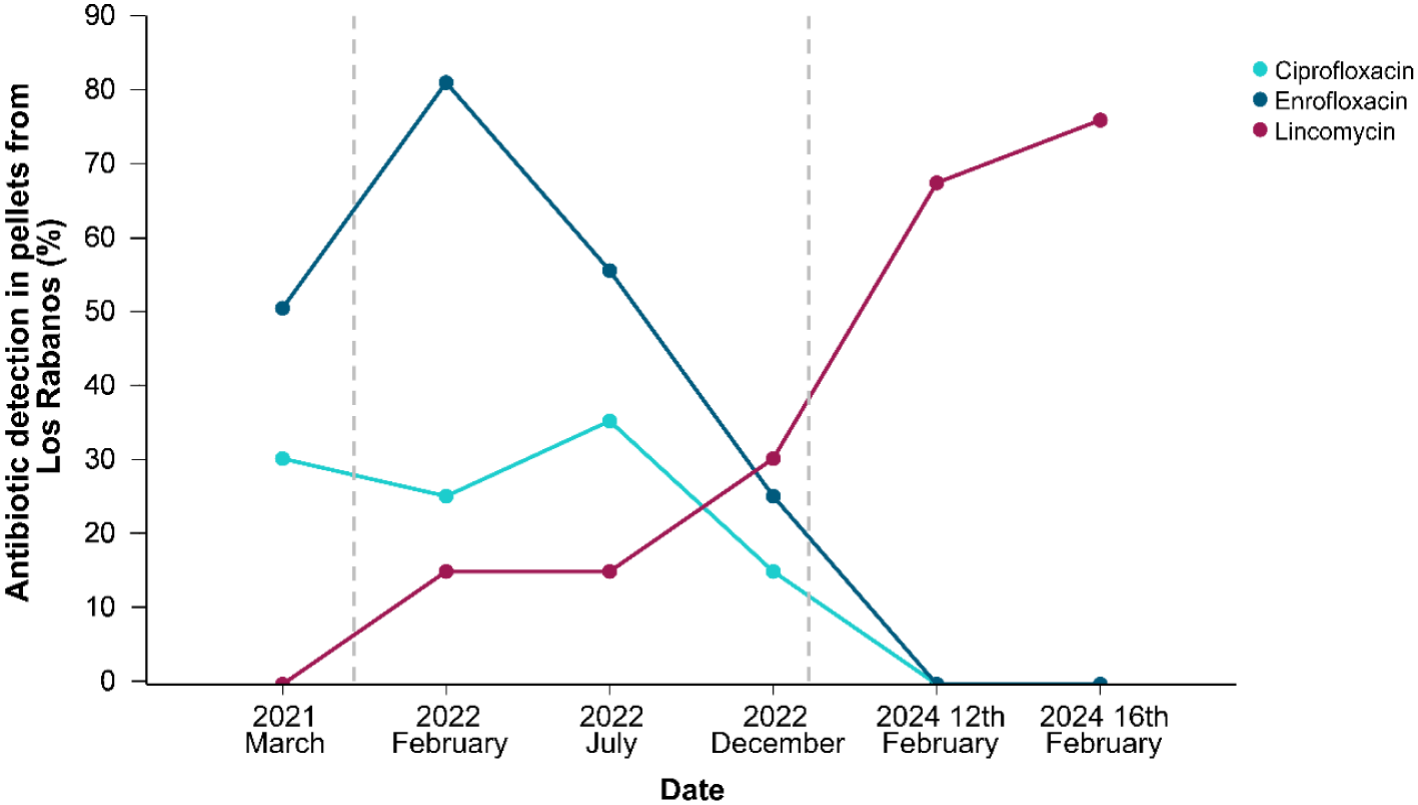
Temporal variation in the prevalence of ciprofloxacin, enrofloxacin, and lincomycin in Los Rábanos pellets: 20 pellets were sampled in all cases except for 12th of February 2024 (*n* = 15).

Pellets are formed in large raptors over periods of 2–3 days.^50^ Regarding feeding trials done in Cape vultures (*Gyps coprotheres*) to study energetic requirements, food ingestion events occur every 1–3 days, which aligns with GPS/ACC data on free-ranging griffons.^51,52^ Considering the pharmacokinetics of some of these veterinary drugs in *Gyps* vultures, the half-life of elimination varies widely depending on the compounds and administration route, ranging between 0.33 and 18.7 h for NSAIDs in *Gyps* vultures under experimental conditions.^49^ In other raptors treated with enrofloxacin orally, the half-life was also high (>18 h), even though absorption from the intestinal tract occurred after 2–8 h.^53^ This implies that vultures may consume different carcasses on consecutive days without fully eliminating pharmaceuticals acquired from previous meals. This hypothesis supports the fact that we find pharmaceuticals in pellets formed exclusively by wild animal remains. Besides, pharmaceuticals detected in pellets could represent a nonabsorbed fraction of the compound present in the medicated carcass or reflect partial elimination through gastric secretion. Supporting this hypothesis, in broiler chickens, pharmaceuticals administered with pasty feed can remain 3–20 h in the crop before passing to the stomach, in contrast to pharmaceutical administration through drinking water.^54^ This suggests that pharmaceuticals embedded in an organic matrix might remain longer in birds’ crops, increasing their likelihood of being incorporated into pellets during their formation. Even so, we assume a bias may exist when interpreting the levels found in pellets regarding the exact time of pellet formation and time elapsed since the collection of the sample are not available. This issue should be addressed under experimental conditions to perform a deeper interpretation of the levels found in pellets from the field.

### Conservation Implications

Several pharmaceuticals were detected in avian scavenger pellets for the first time. Most of the compounds analyzed in this study are stable enough to be detected even after one month under outdoor conditions in this matrix. These findings underscore the value of the method developed here as a noninvasive monitoring tool capable of detecting pharmaceutical prevalence values comparable to those observed in wild vultures and livestock carcasses available for their feeding. Our results confirm that vultures in Spain are exposed to a wide variety of pharmaceuticals, which is consistent with previous findings that analyzed other biological matrices like plasma or tissues from wild avian scavengers. Moreover, the pharmaceutical prevalence in pellets from griffon vultures is positively associated with diet composition. The management intensity and type of farming supplying carcass dumping sites appear to be key drivers of pharmaceutical exposure. Pellets containing pig remains, which are typically linked to intensive management, showed higher prevalences of pharmaceuticals in contrast to those formed by sheep wool, which are species linked to extensive husbandry. Furthermore, despite awareness of the relative toxicity of NSAIDs, flunixin continues to be detected within the vulture’s trophic level. Therefore, in the interest of safeguarding wild vulture populations, we recommend improving the regulations on carcasses supplied for vulture feeding, together with enhanced communication among stakeholders to prevent medicated livestock carcasses from being delivered to supplementary feeding stations and the use of already registered and safe NSAIDs like meloxicam and tolfenamic acid.^55,56^ Mitigation measures should be implemented in line with priority safety testing of such drugs on *Gyps* vultures to address the knowledge gap regarding toxicity thresholds and mechanisms of action. This information could be used to assess the need to ban the use of flunixin in regions where vulture populations are still threatened and to prevent exposure.

## Supplementary Material



## Notes

Additional details on sampling procedures, quality control of the analytical method, including intraday and interday recoveries, limits of detection and quantification (LODs, LOQs), linearity of calibrations performed in sample analysis, percentage of pharmaceuticals detected in the stability test, and details on statistical analysis (PDF)
