# Supplementary material for: Tracing Veterinary Pharmaceuticals through Regurgitated Pellets: A Noninvasive Monitoring Approach to Assess Exposure in Avian Scavengers
Source: Environ Sci Technol. 2026 Jun 18;60(25):17696–705. doi: 10.1021/acs.est.6c01303 (PMC13325845; doi:10.1021/acs.est.6c01303)
Supplement: Supplementary file 1 [file es6c01303_si_001.pdf]

SUPPORTING INFORMATION

Tracing veterinary pharmaceuticals through regurgitated pellets: a non-invasive monitoring approach to assess exposure in avian scavengers

Nelly Arlot[1], Marta Herrero-Villar[1*], Rafael Mateo[1,2]

[1]Institute for Game and Wildlife Research-IREC, CSIC-UCLM-JCCM, Ronda de Toledo s/n, 13071 Ciudad Real, Spain

[2]Institute for Environmental Assessment and Water Research (IDAEA-CSIC), Jordi Girona 18, 08034 Barcelona, Spain

[*]Corresponding author: marta.herrero@uclm.es

SUMMARY CONTENT

Table S1. Total pellets analysed for pharmaceuticals including selected sites, province, region and year of sampling.

Table S2. Intraday and interday recoveries, limit of detection (LOD), limit of quantification and linearity of calibrations (R^2) of the monitored pharmaceuticals.

Table S3. Percentage of pharmaceutical detected in spiked pellets up to 28 days of storage at outdoor environmental conditions in comparison to the amount detected at time 0.

Table S4. Results of the main Generalized Linear Models used to study the determinants of the presence and concentrations of pharmaceuticals in the griffon vulture pellets, final models selected appear in italics and grey background.

Table S5. Comparison of the competing GLM models using information-theoretic criteria (AICc, ΔAICc, Akaike weights) used to study the determinants of the presence and concentrations of pharmaceuticals in the griffon vulture pellets.

Table S1. Total pellets analysed for pharmaceuticals including selected sites, province, region and year of sampling.

Region	Province	Site	Year	N
Aragon	Zaragoza	Calatayud	2022	10
		Illueca	2022	10
		Morata del Jalón	2024	13
	Teruel	La Mata de los Olmos	2022	16
		Perales de Alfambra	2022	4
Castilla-La Mancha	Guadalajara	Peralejos de las Truchas	2024	4
		Pobo de Dueñas	2024	10
		Ventosa	2024	10
Castilla León	Burgos	Caleruega	2024	10
		Nebreda	2024	5
		Huerta de Rey	2024	5
	Soria	Los Rábanos	2021 (March)	20
			2022 (February)	20
			2022 (July)	20
			2022 (December)	20
			2024 (12th February)	15
			2024 (16th February)	20
		Berlanga del Duero	2021	20
			2024	20
La Rioja	La Rioja	Igea	2024	20
Navarra	Navarra	Murillo	2024	20
**Total**				**292**

Table S2. Intraday and interday recoveries, limit of detection (LOD), limit of quantification and linearity of calibrations (R^2) of the monitored pharmaceuticals.

Concentrations (ng/mL)	Intraday		Inter-day		LOD (ng/g)	LOQ (ng/g)	R^2
	Mean recovery %	RSD %	Mean recovery %	RSD %			
**Caffeine**							
**100**	111	2.8	103	16.9	0.83	2.76	0.9872
**50**	108	7.1	97	37.3			
**25**	108	8.0	123	5.1			
**Carprofen**							
100	83	25.5	99	24.7	1.493	4.978	0.9914
50	74	52.7	86	14.4			
25	86	50.7	116	21.7			
**Chloramphenicol**							
100	101	12.4	98	18.1	0.854	2.845	0.9909
50	103	12.5	102	23			
25	85	8.4	95	26.4			
**Chlortetracycline**							
100	98	3.8	84	27.4	0.538	1.795	0.9850
50	75	7.3	81	17.3			
25	81	6.1	80	20.5			
**Ciprofloxacin**							
100	93	11.7	72	13.7	0.031	0.103	0.9937
50	77	3.1	65	9.4			
25	72	36.9	75	29.2			
**Clindamycin**							
100	108	1.3	98	26.2	0.682	2.274	0.9923
50	100	10.7	94	30.8			
25	96	7	93	27.1			
**Cloxacillin**							
100	104	5.3	96	27.7	0.238	0.792	0.9933
50	102	5.7	78	17.7			
25	102	10.0	87	27.9			
**Danofloxacin**							
100	100	5.9	77	17.8	0.031	0.102	0.9859
50	77	4.8	71	13.8			
25	77	23.1	73	28.4			
**Diclofenac**							
100	115	3.0	103	22.6	0.250	0.834	0.9959
50	108	12.8	100	13			
25	105	12.9	116	19.3			
**Doramectin**							
100	116	1.2	96	28.5	1.212	4.041	0.9766
50	107	11.7	94	31.5			
25	113	19.2	113	21.7			
**Doxycycline**							
100	111	3.7	86	31.4	0.047	0.156	0.9903
50	98	14.7	80	30.1			
25	118	27.1	89	26			
**Enrofloxacin**							
100	109	5.3	82	17.7	0.009	0.029	0.9958
50	101	17.9	71	18			

Concentrations (ng/mL)	Intraday		Inter-day		LOD (ng/g)	LOQ (ng/g)	R^2
	Mean recovery %	RSD %	Mean recovery %	RSD %			
25	91	40.4	76	31.1			
**Eprinomectin**							
100	95	16.4	104	25.5	0.187	0.623	0.9810
50	86	39.0	92	25.3			
25	83	21.3	111	19.2			
**Erythromycin**							
100	100	2.2	102	16.9	0.082	0.273	0.9885
50	100	6.2	105	27.5			
25	93	5.5	96	17			
**Florfenicol**							
100	107	3.2	100	23.8	0.559	1.862	0.9844
50	114	8.2	93	12.8			
25	104	7.0	94	20			
**Flumequine**							
100	112	5.4	101	16.9	0.006	0.020	0.9940
50	97	10.2	93	4.6			
25	111	6.0	93	22.6			
**Flunixin**							
100	111	3.6	106	20.2	0.013	0.043	0.9961
50	111	6.0	104	23.9			
25	95	9.1	111	19.5			
**Gamithromycin**							
100	116	2.6	91	28	0.014	0.048	0.9929
50	100	10.1	89	34.7			
25	89	13.1	89	32.1			
**Hydroxyetil salicylate**							
100	115	2.8	97	25	0.005	0.016	0.9973
50	112	7.1	99	23.3			
25	106	7.4	93	24.4			
**Indomethacin**							
100	88	12.2	104	20.7	5.666	18.888	0.9897
50	86	7.4	111	8.7			
25	109	7.6	108	18.2			
**Ivermectin**							
100	94	12.8	93	20.8	4.516	15.054	0.9765
50	75	8.9	107	19.1			
25	89	5.8	100	20.2			
**Ketoprofen**							
100	114	1.7	102	20.1	0.029	0.098	0.9953
50	109	6.1	96	20.2			
25	98	8.4	106	19.4			
**Lincomycin**							
100	117	2.1	95	31.1	0.002	0.006	0.9953
50	107	7.1	81	21.3			
25	111	7.7	89	23.8			
**Marbofloxacin**							
100	92	7.7	75	20.1	0.001	0.005	0.9868
50	73	3.9	71	7.5			
25	67	38.9	75	28.9			
**Meloxicam**							
100	112	4.7	105	22.3	0.002	0.007	0.9949

Concentrations (ng/mL)	Intraday		Inter-day		LOD (ng/g)	LOQ (ng/g)	R^2
	Mean recovery %	RSD %	Mean recovery %	RSD %			
50	109	6.5	89	24.7			
25	104	3.4	100	19.9			
**Nalidixic Acid**							
100	114	2.8	94	20.1	0.071	0.235	0.9956
50	111	7.5	86	14			
25	95	3.5	89	27.8			
**Naproxen**							
100	116	3.1	102	18.2	0.060	0.200	0.9954
50	110	4.4	87	8.3			
25	105	7.1	105	11.7			
**Nimesulide**							
100	117	1.6	108	20.5	0.073	0.243	0.9907
50	100	10.6	99	18.9			
25	99	12.8	103	19.2			
**Oxacillin**							
100	105	4.6	86	24.4	0.297	0.989	0.9945
50	102	8.2	89	31.5			
25	98	11.9	84	32			
**Penicillin G**							
100	82	7.4	82	25.5	0.234	0.779	0.9940
50	109	7.8	88	30.4			
25	95	9.1	79	26.7			
**Penicillin V**							
100	86	12.4	88	29.9	0.690	2.298	0.9898
50	99	11.0	77	17.1			
25	87	30.2	98	28.2			
**Phenylbutazone**							
100	87	19.0	97	25.1	1.790	5.966	0.9965
50	94	19.7	79	16.3			
25	103	14.5	87	17.9			
**Scopolamine**							
100	102	10.4	87	17.7	0.013	0.045	0.9944
50	93	4.3	79	15.2			
25	79	29.2	73	28.5			
**Spiramycin**							
100	117	1.3	98	20.7	0.101	0.336	0.9925
50	109	4.3	93	26.3			
25	103	23.8	86	20.9			
**Sulfadiazine**							
100	94	5.7	91	31.3	0.002	0.007	0.9954
50	106	9.4	90	30.1			
25	99	12.3	88	24.1			
**Sulfadimidine**							
100	102	8.0	93	24.3	0.006	0.020	0.9924
50	87	12.7	97	25.5			
25	82	8.5	90	28.1			
**Sulfadoxine**							
100	100	8.4	100	23.2	0.001	0.004	0.9918
50	112	8.5	100	33.7			
25	105	8.0	95	24.5			
**Sulfamethoxypyridazine**							
100	106	3.4	93	24.7	0.001	0.002	0.9932

Concentrations (ng/mL)	Intraday		Inter-day		LOD (ng/g)	LOQ (ng/g)	R^2
	Mean recovery %	RSD %	Mean recovery %	RSD %			
50	109	8.9	97	25.9			
25	96	12.9	88	21.7			
**Sulfamethoxazole**							
100	114	2.2	91	23.4	0.001	0.003	0.9940
50	109	9.8	88	24.7			
25	107	13.6	91	23			
**Suxibuzone**							
100	113	6.3	109	15.2	0.125	0.416	0.9943
50	95	7.5	109	20.5			
25	102	7.4	104	11.4			
**Tetracycline**							
100	98	4.9	80	16.5	0.180	0.601	0.9938
50	98	8.4	76	11			
25	94	10.9	81	20.2			
**Thiamphenicol**							
100	112	6.0	95	28.3	0.031	0.104	0.9957
50	101	6.3	91	28.9			
25	92	1.4	96	25.0			
**Tiamulin**							
100	108	3.6	100	28.1	0.005	0.016	0.9949
50	107	4.8	93	33.7			
25	111	5.3	93	23.6			
**Tilmicosin**							
100	105	5.7	106	18.4	0.225	0.752	0.9912
50	112	4.9	120	12.6			
25	99	4.3	101	22.0			
**Tolfenamic acid**							
100	99	2.5	95	16.7	0.011	0.037	0.9918
50	105	5.9	88	15.3			
25	101	2.1	93	19.2			
**Trimethoprim**							
100	104	2.9	94	31.3	0.001	0.004	0.9916
50	97	4.6	92	23.0			
25	100	8.8	91	29.6			
**Tulathromycin**							
100	106	11.5	88	30.0	0.107	0.357	0.9924
50	103	7.2	87	32.5			
25	84	8.8	72	27.2			

Table S3. Percentage of pharmaceutical detected in spiked pellets up to 28 days of storage at outdoor environmental conditions in comparison to the amount detected at time 0.

	Day													
	0		1		2		4		7		14		28	
**Group/Pharmaceutical**	**Mean**	**SD**	**Mean**	**SD**	**Mean**	**SD**	**Mean**	**SD**	**Mean**	**SD**	**Mean**	**SD**	**Mean**	**SD**
**ANTIMICROBIALS**														
Chlortetracycline	100	17.1	67.9	7.4	67.7	16.3	65.0	9.9	68.4	25.1	69.2	15.0	43.6	8.0
Ciprofloxacin	100	13.4	93.8	8.8	91.2	19.3	90.7	7.0	94.4	24.2	84.5	11.6	65.8	11.4
Clindamycin	100	17.1	47.3	10.3	59.2	15.1	51.3	6.3	79.6	24.4	90.0	26.0	66.0	18.6
Cloxacillin	100	23.6	54.9	9.9	59.4	10.2	64.8	7.4	103.1	38.3	114.5	39.0	81.9	27.0
Danofloxacin	100	11.4	83.9	4.6	80.6	10.1	86.1	7.3	97.4	32.3	93.5	12.8	77.1	14.1
Doxycycline	100	19.9	87.1	8.5	81.3	7.0	71.6	4.7	86.6	26.7	90.4	19.2	49.8	9.6
Enrofloxacin	100	15.2	82.4	10.8	81.9	9.2	83.7	5.6	80.7	13.6	78.9	13.0	66.3	12.8
Erythromycin	100	19.1	57.0	11.1	48.2	8.4	47.3	5.5	68.7	29.7	74.3	21.6	62.6	15.9
Florfenicol	100	16.6	79.9	11.3	85.5	11.9	88.8	9.5	121.8	49.4	117.7	37.8	110.3	15.4
Flumequine	100	26.2	69.6	6.5	65.2	9.3	64.7	5.3	86.1	28.4	84.2	19.5	66.1	16.7
Gamithromycin	100	10.8	73.3	4.9	76.6	12.1	76.8	6.3	105.8	33.4	116.5	18.3	114.8	10.0
Lincomycin	100	11.6	67.5	7.0	68.6	12.4	61.7	6.9	91.6	30.0	103.7	21.1	82.3	17.7
Marbofloxacin	100	15.3	80.9	8.3	74.1	16.4	72.4	11.3	71.3	15.1	75.7	5.3	46.9	9.2
Nalidixic Acid	100	24.8	84.4	13.0	76.4	11.7	83.3	9.0	121.1	58.0	112.3	32.3	100.6	25.8
Oxacillin	100	16.7	60.6	9.1	69.8	8.9	64.7	10.1	106.4	21.8	111.1	23.4	106.9	16.4
Penicillin G	100	20.4	58.5	9.7	57.1	14.4	56.5	4.9	84.5	26.6	86.6	26.5	56.5	14.2
Penicillin V	100	21.9	78.4	3.1	79.0	7.1	76.5	5.0	107.6	32.1	116.6	18.1	123.3	15.5
Spiramycin	100	15.0	66.9	8.6	65.8	18.2	61.1	6.2	79.2	21.6	89.7	25.3	50.9	13.1
Sulfadiazine	100	19.6	62.4	5.7	68.2	12.4	70.0	15.8	130.4	52.3	128.6	58.5	104.4	32.1
Sulfadimidine	100	21.7	55.5	6.4	60.8	11.8	50.2	4.7	89.1	29.7	93.9	24.8	74.1	11.4
Sulfadoxine	100	17.2	57.8	6.8	62.9	9.8	60.0	8.0	101.0	37.0	100.9	37.3	73.9	22.7
Sulfamethoxypyridazine	100	22.0	57.4	6.2	63.9	10.3	61.6	10.7	105.9	36.2	116.6	27.4	83.6	23.1
Sulfametoxazole	100	23.6	67.3	9.2	71.2	12.9	68.6	12.6	114.1	40.3	112.5	42.4	99.5	13.9
Tetracycline	100	15.4	75.2	3.0	57.0	9.3	57.3	6.8	85.0	29.5	83.6	25.2	53.9	8.7
Thiamphenicol	100	22.0	77.7	10.6	89.0	19.2	95.8	27.6	141.8	72.3	133.5	48.5	147.7	30.8
Tiamulin	100	20.5	48.2	6.3	48.5	15.5	48.7	12.1	82.2	29.9	93.2	41.5	66.1	14.5
Tilmicosin	100	15.4	68.8	4.0	65.0	6.9	63.6	5.4	81.1	23.8	82.6	15.9	61.8	6.1
Trimethoprim	100	18.0	73.4	5.5	72.7	11.3	75.1	6.3	110.7	38.6	106.3	24.0	91.4	17.3
Tulathromycin	100	23.0	138.2	14.5	134.2	20.0	115.2	22.5	110.1	20.3	121.6	36.9	100.2	22.9
**NSAID**														
Diclofenac	100	7.9	38.1	5.9	34.9	13.6	30.1	2.8	34.9	4.7	30.7	10.4	18.3	2.8
Flunixin	100	3.0	88.5	6.7	90.3	16.4	85.1	4.0	91.0	6.4	93.0	7.1	82.4	5.4
Hydroxyetil salicylate	100	2.4	80.3	2.8	70.2	15.4	55.5	1.5	52.6	3.9	48.2	5.9	33.5	3.6
Ketoprofen	100	5.9	64.1	6.3	62.3	14.1	52.7	3.4	56.9	8.3	53.7	14.5	35.1	7.1
Meloxicam	100	3.2	37.3	8.0	34.5	12.1	30.1	3.1	31.2	4.3	34.7	3.5	23.5	4.9
Naproxen	100	14.9	56.1	6.6	52.3	8.6	42.8	4.5	38.8	3.9	38.8	2.0	24.5	6.7
Nimesulide	100	23.2	39.6	6.7	41.5	24.1	40.4	11.0	42.5	8.2	43.6	5.5	26.9	2.9
Suxibuzone	100	9.3	94.0	11.0	95.2	16.9	97.4	9.8	107.1	8.9	99.8	8.0	85.2	2.2
Tolfenamic acid	100	3.7	67.5	5.0	54.7	11.5	51.5	1.6	45.9	8.2	38.7	1.7	30.1	4.4
**OTHER**														
Scopolamine	100	11.8	77.6	11.7	65.3	17.0	53.7	8.0	34.7	7.4	29.9	5.1	22.3	4.0

Table S4. Results of the main Generalized Linear Models used to study the determinants of the presence and concentrations of pharmaceuticals in the griffon vulture pellets, final models selected appear in italics and grey background.

MODEL 1A. Dependent variable: Pharmaceutical presence / Independent variables: Region + Type of prey + Year (AIC = 102.9)								
Variables		β	Standard error	95% CI		Statistical test		
				Lower	Upper	χ^2 Wald	d.f.	p-value
Intercept		-3.520	0.9205	-5.324	-1.716	14.620	1	0.000
Region	Aragon	2.365	0.8311	0.736	3.994	8.097	1	0.004
	Castilla-La Mancha	1.001	0.9026	-0.768	2.770	1.231	1	0.267
	Castilla y León	2.378	0.7848	0.840	3.916	9.183	1	0.002
	La Rioja	1.143	0.9422	-0.704	2.989	1.471	1	0.225
	Navarra	0	.	.	.	.	.	.
Type of prey	Domestic	1.322	0.5402	0.264	2.381	5.993	1	0.014
	Mixed	0.883	0.5656	-0.226	1.991	2.435	1	0.119
	Wild	0	.	.	.	.	.	.
Year	2021	-0.371	0.4006	-1.156	0.414	0.859	1	0.354
	2022	0.456	0.3086	-0.149	1.061	2.187	1	0.139
	2024	0	.	.	.	.	.	.
**MODEL 1B. Dependent variable: Pharmaceutical presence / Independent variables: Region + Type of prey (AIC = 103.2)**								
Variables		β	Standard error	95% CI		Statistical test		
				Lower	Upper	χ^2 Wald	d.f.	p-value
Intercept		-3.668	0.917	-5.466	-1.871	16.001	1	<0.001
Region	Aragon	2.695	0.803	1.121	4.268	11.268	1	<0.001
	Castilla-La Mancha	1.014	0.903	-0.755	2.783	1.262	1	0.261
	Castilla y León	2.478	0.768	0.974	3.983	10.421	1	0.001
	La Rioja	1.170	0.943	-0.677	3.018	1.541	1	0.214
	Navarra	0	.	.	.	.	.	.
Type of prey	Domestic	1.471	0.534	0.424	2.518	7.582	1	0.006
	Mixed	0.982	0.561	-0.118	2.081	3.061	1	0.080
	Wild	0	.	.	.	.	.	.
**MODEL 1C. Dependent variable: Pharmaceutical presence / Independent variables: Region + Type of prey (AIC = 104.1)**								
Variables		β	Standard error	95% CI		Statistical test		
				Lower	Upper	χ^2 Wald	d.f.	p-value
Intercept		-3.574	0.9189	-5.375	-1.773	15.129	1	0.000
Region	Aragon	2.900	0.8183	1.296	4.504	12.559	1	0.000
	Castilla-La Mancha	1.015	0.9027	-0.754	2.784	1.264	1	0.261
	Castilla y León	2.510	0.7734	0.994	4.026	10.531	1	0.001
	La Rioja	1.161	0.9423	-0.686	3.008	1.519	1	0.218
	Navarra	0	.	.	.	.	.	.

Type of prey	Domestic	1.377	0.5375	0.324	2.430	6.564	1	0.010
	Mixed	0.875	0.5664	-0.235	1.985	2.388	1	0.122
	Wild	0	.	.	.	.	.	.
Season	Spring	-0.416	0.3171	-1.038	0.205	1.722	1	0.189
	Summer	0.516	0.5088	-0.481	1.513	1.028	1	0.311
	Winter	0	.	.	.	.	.	.
MODEL 2A. Dependent variable: Pharmaceutical presence / Independent variables: Year + Pig presence (AIC = 181.4)								
Variables		β	Standard error	95% CI		Statistical test		
				Lower	Upper	χ2 Wald	d.f.	p-value
Intercept		-0.114	0.2079	-0.522	0.293	0.302	1	0.582
Year	2021	-0.115	0.3802	-0.860	0.630	0.092	1	0.762
	2022	0.825	0.2806	0.275	1.375	8.652	1	0.003
	2024	0	.	.	.	.	.	.
Pig presence	Yes	-1.057	0.2706	-1.587	-0.526	15.244	1	0.000
	No	0	.	.	.	.	.	.
**MODEL 2B. Dependent variable: Pharmaceutical presence / Independent variables: Region + Pig presence (AIC = 183.3)**								
Variables		β	Standard error	95% CI		Statistical test		
				Lower	Upper	χ2 Wald	d.f.	p-value
Intercept		-1.461	0.7933	-3.016	0.094	3.392	1	0.066
Region	Aragon	1.970	0.8176	0.368	3.573	5.808	1	0.016
	Castilla-La Mancha	0.612	0.9098	-1.171	2.395	0.452	1	0.501
	Castilla y León	1.663	0.7882	0.118	3.208	4.451	1	0.035
	La Rioja	0.768	0.9353	-1.065	2.601	0.675	1	0.411
	Navarra	0	.	.	.	.	.	.
Pig presence	Yes	-0.789	0.2917	-1.361	0-.217	7.316	1	0.007
	No	0						
**MODEL 2C. Dependent variable: Pharmaceutical presence / Independent variables: Region + Pig presence + Year (AIC = 181.8)**								
Variables		β	Standard error	95% CI		Statistical test		
				Lower	Upper	χ^2 Wald	d.f.	p-value
Intercept		-1.506	0.7944	-3.063	0.051	3.592	1	0.058
Region	Aragon	1.655	0.8427	0.004	3.307	3.859	1	0.049
	Castilla-La Mancha	0.629	0.9091	-1.153	2.411	0.478	1	0.489
	Castilla y León	1.615	0.8034	0.041	3.190	4.042	1	0.044
	La Rioja	0.771	0.9348	-1.061	2.603	0.680	1	0.410
	Navarra	0	.	.	.	.	.	.
Pig presence	Yes	-0.740	0.2953	-1.319	-0.162	6.285	1	0.012
	No	0	.	.	.	.	.	.
Year	2021	-0.415	0.3973	-1.194	0.364	1.090	1	0.296
	2022	0.512	0.3069	-0.090	1.113	2.780	1	0.095

	2024	0	.	.	.	.	.	.
**MODEL 3. Dependent variable: Pharmaceutical concentration (log-transformed) / Independent variables: Region + Sheep presence (AIC = 890.0)**								
Variables		β	Standard error	95% CI		Statistical test		
				Lower	Upper	χ^2 Wald	d.f.	p-value
Intercept		-0.180	0.2816	-0.732	0.372	0.407	1	0.524
Region	Aragon	0.862	0.2851	0.303	1.421	9.145	1	0.002
	Castilla-La Mancha	0.160	0.3320	-0.491	0.811	0.232	1	0.630
	Castilla y León	0.638	0.2562	0.135	1.140	6.194	1	0.013
	La Rioja	0.432	0.3428	-0.240	1.104	1.589	1	0.207
	Navarra	0	.	.	.	.	.	.
Sheep presence	Yes	0.309	0.1594	-0.003	0.622	3.768	1	0.052
	No	0	.	.	.	.	.	.

Table S5. Comparison of the competing GLM models using information-theoretic criteria (AICc, ΔAICc, relative likelihood, Akaike weights) used to study the determinants of the presence and concentrations of pharmaceuticals in the griffon vulture pellets.

Models	AICc	ΔAICc	Likelihood (L_i)	Weight (w_i)
**MODEL 1. Dependent variable: Pharmaceutical presence**				
Region + Specie + Year	102.95	0.00	1.00	0.41
Region + Specie	103.20	0.26	0.88	0.36
Region + Specie + Season	104.14	1.19	0.55	0.23
**MODEL 2. Dependent variable: Pharmaceutical presence**				
Pig + Year	181.42	0.00	1.00	0.45
Pig + Region +Year	181.77	0.35	0.84	0.38
Pig + Region	183.34	1.91	0.38	0.17
**MODEL 3. Dependent variable: Pharmaceutical concentration (log-transformed)**				
Sheep + Region	889.99	0.00	1.00	0.54
Sheep + Pig + Region	892.07	2.09	0.35	0.19
Pig+ Region	893.46	3.48	0.18	0.10
Sheep + Year + Region	893.56	3.57	0.17	0.09
Sheep + Year	893.71	3.72	0.16	0.08
